# SinoMedminer: an R package and shiny application for mining and visualizing traditional Chinese medicine herbal formulas

**DOI:** 10.1186/s13020-025-01127-9

**Published:** 2025-06-06

**Authors:** Wenchao Dan, Xinyuan Guo, Guangzhong Zhang, Hui Zhang, Jin Liu, Qiushuang Li, Yang Chen, Qingyong He

**Affiliations:** 1 Department of Cardiology, Guang’anmen Hospital, China Academy of Chinese Medical Sciences, Xicheng District, Beixian Pavilion Street No. 5, Beijing, 100053 China; 2 Department of Radiation Oncology, National Cancer Center/National Clinical Research Center for Cancer/Cancer Hospital, Chinese Academy of Medical Sciences and Peking Union Medical College, Beijing, 100021 China; 3 Beijing Hospital of Traditional Chinese Medicine, Capital Medical University, Beijing, 100010 China; 4 Center of Clinical Evaluation and Analysis, The First Affiliated Hospital of Zhejiang Chinese Medical University, Hangzhou, 310006 China; 5 Department of Traditional Chinese Medicine, First Clinical Medical College, Shaanxi University of Chinese Medicine, Xianyang, 712046 Shanxi China

**Keywords:** Traditional Chinese medicine, Data mining, R language, Shiny application development

## Abstract

This study addresses limitations of mainstream approaches in traditional Chinese medicine (TCM) data mining by developing the SinoMedminer R package and its Shiny web application. The R package's core functionalities include data cleaning, transformation, TCM attribute statistics, association rule exploration and analysis, clustering analysis, co-occurrence network analysis, formula similarity analysis, formula identification, and dosage analysis. This package enables efficient project analyses without requiring complex coding. The accompanying Shiny web application provides an interactive, menu-driven interface for users without programming knowledge. SinoMedminer combines the computational power of a programming language with user-friendly accessibility, significantly enhancing the efficiency and standardization of TCM data mining research. A deployed server platform further simplifies access and usability by allowing direct utilization of the Shiny application. By optimizing data processing and analysis workflows, SinoMedminer enhances big data handling capabilities, accelerates research progress and product development, and promotes the integration of digital technologies into TCM research and clinical practice.

## Introduction

Data mining of traditional Chinese medicine (TCM) formulas to identify core herbs or treatment targets is a technical approach that has emerged in the past decade [1, 2]. In our previous research, we highlighted the limitations of existing statistical methods for mining TCM prescriptions and proposed relevant improvements [3]. However, based on our team's experience with dozens of TCM data mining projects, TCM clinical data are often complex, multidimensional datasets that include patient information, diagnoses, syndrome differentiation, symptoms, formula composition, dosages, acupoints, and other relevant factors. The data mining workflow is intricate, involving data entry, data cleaning, data exploration, analysis, and visualization [4]. While structured data are well-suited for data science applications [5], a substantial portion of TCM clinical data exists as unstructured free text [6], particularly in symptom descriptions and diagnostic records, which require specialized techniques for processing unstructured data. Additionally, variations in terminology used to describe the same entities due to differences in region, era, usage, and individual practitioner characteristics pose a significant challenge. Consequently, in-depth mining of TCM prescriptions requires a diverse skill set in data analysis, including expertise in handling both structured and unstructured data.

Currently, data mining of TCM prescriptions primarily relies on three types of tools. The first type comprises relatively well-established TCM data mining platforms, such as the TCM Inheritance Computing Platform, Inheritance Assistance Platform, and Ancient and Modern Medical Records Cloud Platform [7]. These platforms are user-friendly and suitable for small- to medium-scale data analysis. However, they often experience performance degradation, such as computational delays and slow response times, when processing large-scale TCM datasets. Furthermore, the visualization features of these platforms require improvements in terms of aesthetics, as some visualization outputs are not suitable for direct inclusion in high-impact academic publications. For instance, certain visualizations lack the clarity or precision required by top-tier journals. Therefore, while these platforms offer ease of use, their performance and visualization capabilities may constrain their applicability in large-scale, publication-oriented research.

The second type of tool comprises menu-based statistical software, such as SPSS Modeler and JMP [8]. These tools offer the advantage of intuitive interfaces and broad statistical capabilities. However, they present several limitations: (i) susceptibility to computational delays when hardware resources are insufficient; (ii) the requirement for users to independently prepare and clean standardized TCM data, which can be labor-intensive and demands domain-specific expertise; and (iii) limitations in the aesthetic quality of visualizations, which may not meet the standards for publication in high-impact academic journals. Despite their ease of use, these tools are often constrained by hardware limitations and necessitate substantial preprocessing efforts, rendering them less suitable for large-scale TCM data analysis. The third approach utilizes programming languages such as R or Python. However, the inherent complexity of TCM prescription workflows means that a purely programming-based approach can lead to a proliferation of variables, posing challenges for later maintenance.

Several online data analysis platforms for TCM have been developed, such as MicrobeTCM, which analyzes the relationship between Chinese herbs and microorganisms [9], TCMSSD, which supports the standardization of TCM syndromes [10], and ETCM, which explores the connections between Chinese herbs and modern molecular biology networks [11]. However, no application specifically designed for TCM prescription analysis currently exists. Therefore, this study aims to develop an R package, SinoMedminer, and its corresponding Shiny web application, specifically for the analysis of TCM clinical prescriptions. This tool seeks to integrate the advantages of the aforementioned three types of methods while mitigating their limitations, thereby accelerating the progress of TCM data mining research, reducing the time required for translating research outcomes into practical applications, and promoting the advancement of TCM academic research and clinical practice.

## Materials and methods

### Test datasets for SinoMedminer package

To ensure the broad applicability and robustness of the developed R package, this study collected datasets from diverse sources, scales, and historical periods, including hospitals, patents, the China National Knowledge Infrastructure (CNKI), ancient texts, and proprietary Chinese medicines. Additionally, to evaluate the efficiency of the R package, particular attention was paid to distinguishing datasets by their size and scope. Details of a subset of the test datasets are provided in Table 1.

**Table 1:** Test datasets of SinoMedminer package

Datasets	Source	Number of formulations	Key fields
Ward Medical Records 1	Medical records for treating psoriasis in the dermatology department of Beijing Traditional Chinese Medicine Hospital	7178	Herb medicine, syndrome, dosage, dosage form
Ward Medical Records 2	Medical records from the Intensive Care Unit of the Cardiovascular Department at Guang'anmen Hospital, Chinese Academy of Traditional Herb medicine	678	Herb medicine, syndrome, symptoms
Patent Prescription Collection 1	Herbal oral prescriptions for treating psoriasis from the Chinese Patent Announcement Network	485	Herb medicine
Patent Prescription Collection 2	Herbal oral prescriptions for treating shingles from the Chinese Patent Announcement Network	179	Herb medicine
Outpatient Prescription Collection 1	Outpatient records from Master of Traditional Herb medicine Xue Boshou's Pingxintang Clinic	399	Symptoms, herb medicine, dosage, syndrome
Outpatient Prescription Collection 2	Oral prescriptions for treating cough from Zhejiang Provincial Hospital of Traditional Herb medicine	3567	Herb medicine, school
Ancient Prescription Collection 1	Famous prescriptions for treating insomnia from pre-Qin to Qing Dynasty medical masters	309	Herb medicine
Chinese Pharmacopoeia Formulation Collection 1	Kidney-tonifying prescriptions	191	Herb medicine, symptoms
Self-constructed Dataset 1	Randomly generated simulation	357,515	Herb medicine, syndrome

### Built-in standard dataset

In clinical records, identical diagnostic or therapeutic elements should generally not be duplicated. For instance, the symptom list should not contain two instances of"cough," nor should the list of Chinese herbs contain two occurrences of"Phellodendri Cortex." However, compared with tertiary grade-A hospitals specializing in traditional Chinese medicine (TCM), private TCM institutions may possess less robust information systems. Manual transcription of prescriptions from renowned TCM practitioners can also introduce issues such as illegible handwriting or inaccurate herb nomenclature, potentially resulting in duplicate diagnostic or therapeutic entries.

Prior to data analysis, users often need to invest substantial time in data cleaning. To address this challenge, the SinoMedminer package incorporates a built-in standardized dataset. Information on Chinese herbs is derived from the *Chinese Pharmacopoeia* (2020), the"Fourteenth Five-Year Plan" Chinese Medicine textbook, *Traditional Chinese Medicine—Terminology—Part 1: Chinese Materia Medica* (ISO-18662–1:2017), and the Encyclopedia of Traditional Chinese Medicine. Standardized names for diseases, syndromes, therapeutic methods, and symptoms are sourced from the Classification and Codes of Diseases and Syndromes of Traditional Chinese Medicine compiled by the National Administration of Traditional Chinese Medicine, the"Fourteenth Five-Year Plan" Traditional Chinese Medicine Diagnostics textbook, and the Chinese Internal Medicine textbook. Information on acupuncture points is sourced from the"Fourteenth Five-Year Plan" Acupuncture textbook.

### Function development

To enhance the usability of the R package, function names are constructed using the snake_case naming convention, which combines an action and a noun separated by underscores. For example, the function for calculating the properties of traditional Chinese herbs, such as flavor and meridian tropism, is named"calc_property". Functions are categorized into core and non-core functions based on their roles in the workflow. Core functions primarily constitute the key steps in the TCM prescription data mining process. Non-core, or auxiliary, functions are embedded within core functions to support their operation. For instance, the function that calculates the properties of herbs is a core function, whereas a function that sums columns in the standardized dataset and returns a frequency table in descending order is considered an auxiliary function.

### Shiny application design

To accommodate a comprehensive workflow, the Shiny application was developed with two main components: data entry and data analysis. For the data entry module, the ‘shinyjs’ package was utilized to enhance JavaScript interactivity within the Shiny application [12, 13], providing a more dynamic user experience. For the data analysis module, the foundational framework was constructed using the ‘shiny’ package, and the ‘fluidPage’ function was employed to ensure automatic adaptation to varying device screen sizes. Given the complexity of the data analysis functionality, a navigation bar was implemented using the ‘navbarPage’ function, and a multi-page layout was created through the combination of ‘tabsetPanel’ and ‘tabPanel’ functions.

## Results

### Built-in dataset

The dataset includes 8287 collected Chinese herbal names, from which 1005 standardized herbal names were incorporated. Additionally, it contains 2181 relationships regarding herbal properties, such as nature, flavor, and meridian tropism, as well as 928 classifications of herbal efficacy. The dataset also includes 2053 diagnostic terms, 1388 symptom names, 3178 syndrome terms, 1808 therapeutic principles, and 235 classical prescription compositions. Users can utilize the ‘makelookuptable2’ function to rapidly compare their personal datasets with the standardized dataset, generating data that require standardization. For users who require the analysis of rare herbs, the ‘herb_to_add’ function allows them to add their own data based on the example dataset.

### Functions and parameters

**Fig. 1:**
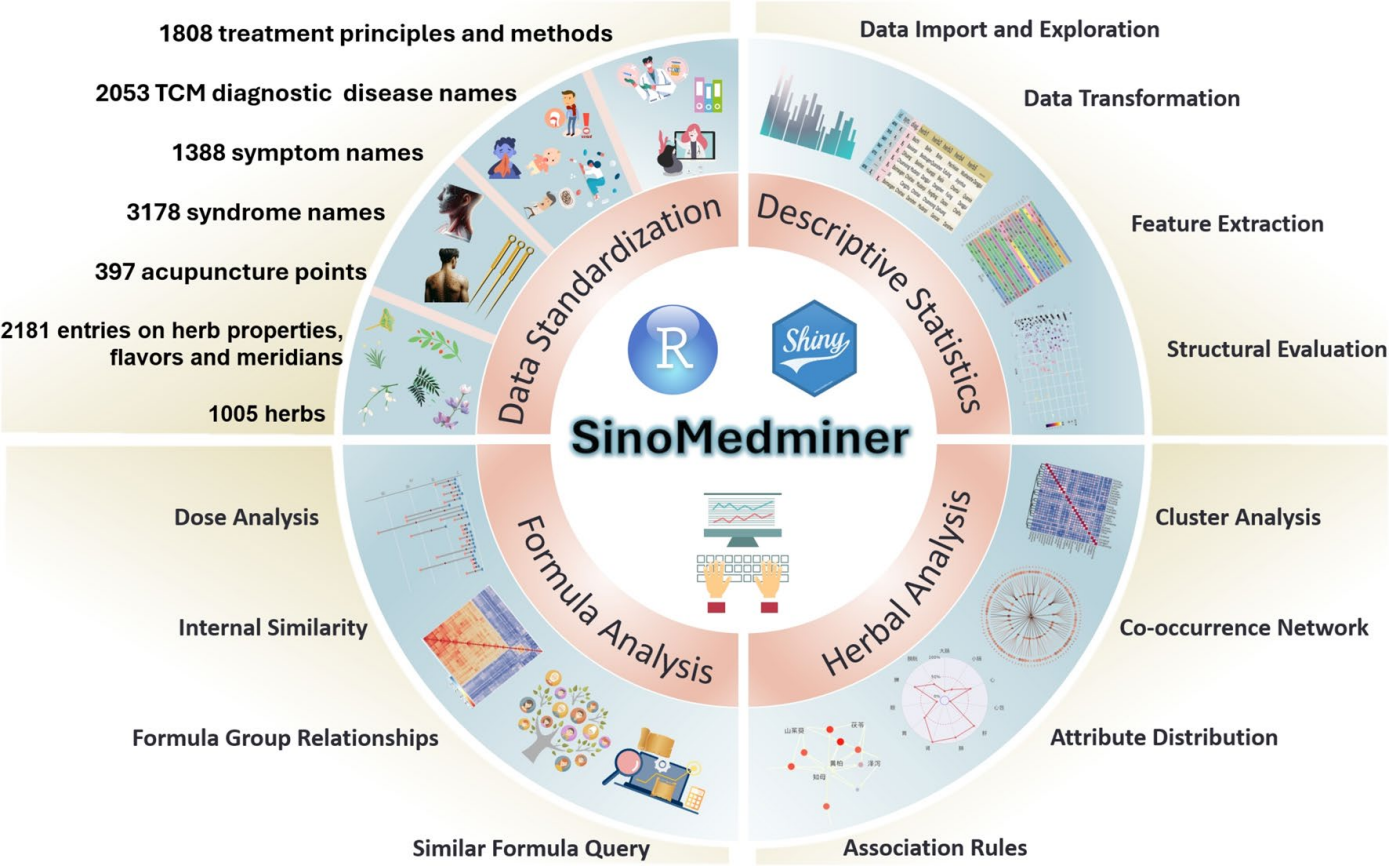
Schematic diagram of the SinoMedminer package's functions

**Fig. 2:**
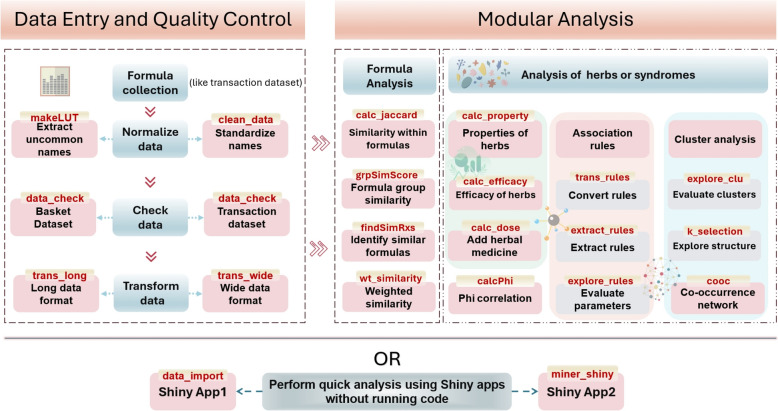
SinoMedminer package workflow diagram

A total of 45 core functions were developed, comprising 15 for data analysis, 19 for visualization, and 11 for data cleaning and transformation. The design of these functions was tailored to reflect the unique characteristics of TCM clinical data. Specifically, nine functions are dedicated to herbal analysis (Table 2), and four functions are dedicated to prescription analysis (Table 3). A schematic representation of the overall functionality of the R package is provided in Fig. 1, while the workflow involving selected core functions in module analysis is shown in Fig. 2. The formula for calculating the similarity coefficient between two groups of prescriptions is presented below.

**Table 2:** Functions of herbal medicine analysis in the SinoMedminer package

Function Name	Functionality
calc_property	Calculates the frequency of the four natures, five flavours, and meridian tropism of TCM herbs. Returns a list containing three data frames with the statistical results for nature, flavor, and meridian tropism, including both weighted and unweighted frequencies
calc_func	Calculates the classification of TCM efficacy
herb_to_add	Compares with the standard dataset to identify any herbs that need to be added
trans_rules	Converts the standard dataset into an association rules object
explore_rules	Sets different combinations of minimum confidence and minimum support, and selects analysis parameters based on the results. Returns a data frame containing the number of association rules, confidence range, support range, and lift. From an application perspective, only results with lift > 1 are retained. Removes -Inf values that occur due to excessively high or low parameter settings
extract_rules	Analyzes association rules and obtains a set of association rules based on the defined parameters
calc_Phi	Calculates the Phi correlation coefficient between herbs and symptoms. Returns a list containing three data frames: the Phi correlation coefficient matrix, the p-value matrix of the Phi correlation, and a matrix of Phi correlation stars (marked with"*"if *p* < 0.05)
cooc	Calculates the co-occurrence frequency of the target field, returning a data frame
explore_cluster	Provides a preliminary evaluation of clustering structures using different methods, including:"ward.D","ward.D2","single","complete","average","centroid","median". Returns seven plots representing the clustering structures of different methods for user evaluation in conjunction with clinical data
k_selection	Uses the votes from 20 different methods to recommend the optimal number of clusters. Provides an objective basis for selecting the number of clusters by counting the recommendation scores for each number of clusters

**Table 3:** SinoMedminer package formula analysis functions

Function name	Description
calc_jaccard	Evaluates the similarity between any two formulas within a formula group. Returns two data frames: one for the Jaccard coefficient matrix and the other for the filtered Jaccard coefficient table
findSimRxs	Identifies formulas similar in composition to the target formula from the standard dataset of formulas
grpSimScore	Assesses the similarity between two formula groups
wt_similarity	Calculates the weighted similarity between the selected core target formula and other formulas. Users need to specify the core formula and predefine the combination of herbs as Monarch, Minister, Assistant, and Envoy. Since there is no established weighting scheme, Zhao Guozhen’s research is used as a reference for now [14]

$$\text{grpSimScore }=$$ $$\frac{1}{\text{n}\times m}$$ $${\Sigma }_{i=1}^{n}{\Sigma }_{j=1}^{m}$$ *J*(*A*_*i*_, *B*_*j*_).

### Visualization functions

The SinoMedminer package includes 15 visualization functions, each prefixed with ‘fig’ to indicate their purpose. These functions are equipped with a range of built-in parameters to enhance the diversity of visual outputs. For instance, the ‘fig_phi’ function, used to generate a heatmap of Phi correlation coefficients, includes eight graphical parameters such as border color, font angle, and clustering tree height. Similarly, the ‘fig_cooc’ function, which visualizes co-occurrence networks, provides nine parameters including network layout, line type, edge color, and node size. An overview of the commonly used visualization functions is presented in Table 4. For detailed information on the parameters of each function, please refer to the respective function's help documentation.

**Table 4:** SinoMedminer package visualization functions

Function name	Description
fig_freq_bar	Counts frequency and plots a frequency bar chart, returning a ggplot2 object. The bar colors gradient based on frequency
fig_exp_rules	Explores the number of rules under different combinations of confidence and support. Returns a bubble chart, where the x-axis represents support, the y-axis represents confidence, and bubble size and color change according to the number of rules
fig_rules_distr	Plots a scatter plot visualizing the distribution of association rules. The x-axis represents support, the y-axis represents confidence, and point size varies with lift, while the number of transactions is represented by color
fig_rules_network/bubble	Plots a network (or bubble) graph of association rules, where node size and color map to selected parameters
fig_cooc	Plots a co-occurrence network graph and returns a ggraph plot object with highly customizable parameters
fig_efficacy	Plots a sunburst chart for the efficacy of Chinese medicine.
fig_guijing/siqi/taste	Plots radar charts for Chinese medicine meridian entry, four properties, and five tastes
fig_jac	Plots a similarity map of prescriptions and returns a list containing the Jaccard matrix, Jaccard relationship table, and heatmap of similarity coefficients using pheatmap

### Shiny applications.

#### Data entry

**Fig. 3:**
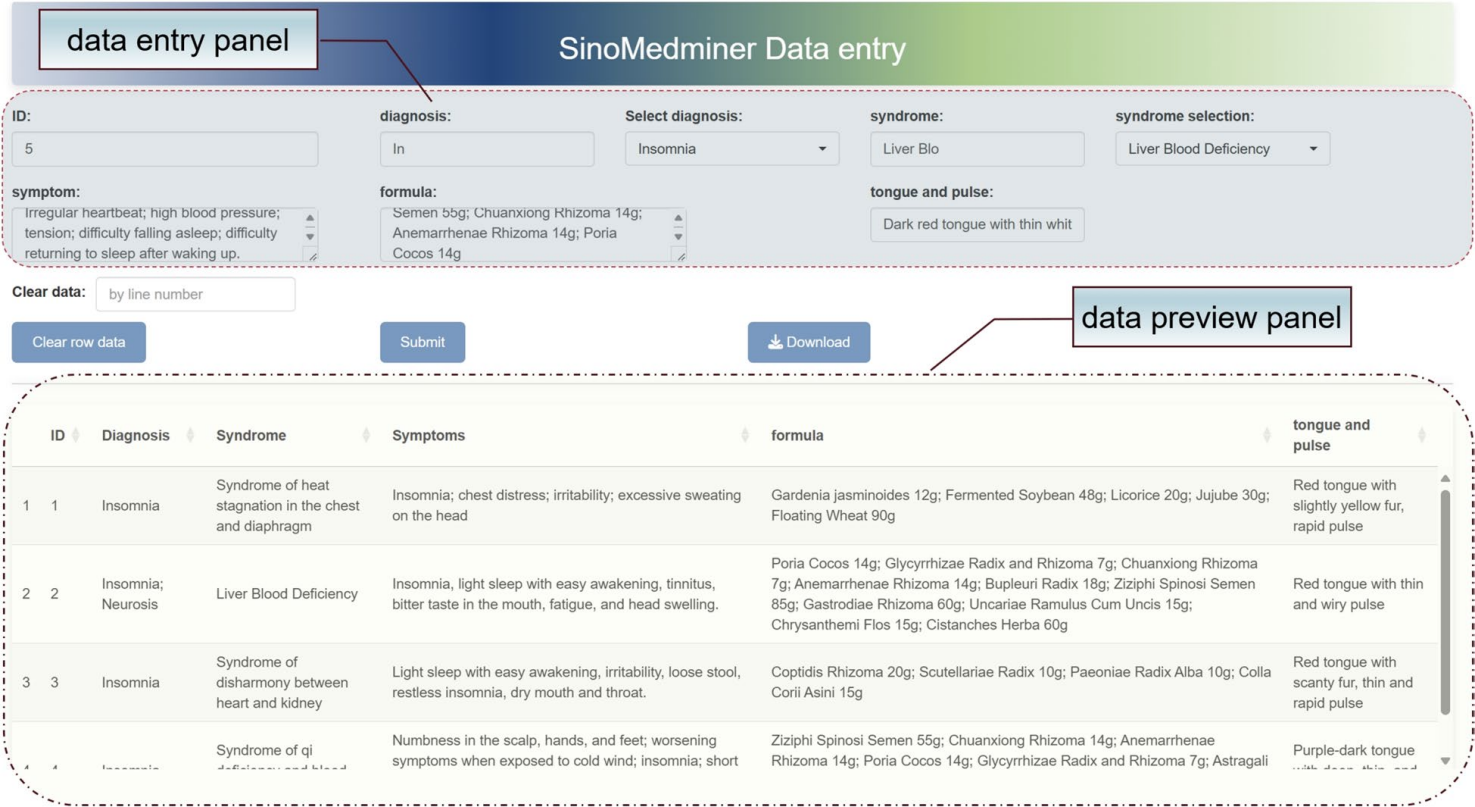
Example of the data entry application in SinoMedminer

Users can directly launch the Shiny data entry application by using the ‘data_import’ function. The Shiny application is designed to collect essential information, including patient ID, diagnosis, syndrome, symptoms, prescription, and tongue/pulse data. The upper section of the application interface is dedicated to data entry, while the lower section provides real-time visualization of the entered data. To enhance the efficiency of data entry, diagnostic and syndrome terms are matched with previously entered information through event listeners, allowing users to select existing entries quickly by pressing "Enter," thereby minimizing repetitive input. JavaScript scripting is employed for key mapping, enabling rapid field switching using the"Tab"key and quick submission of entered data to the table below using the "Ctrl + Enter" shortcut. Upon completion of data entry, users can click the "Download Data" button to export the dataset as an Excel file. An example of the data entry page is shown in Fig. 3. The deployed service platform can also be accessed at http://formulaharmony.com/import.

#### Data analysis

**Fig. 4:**
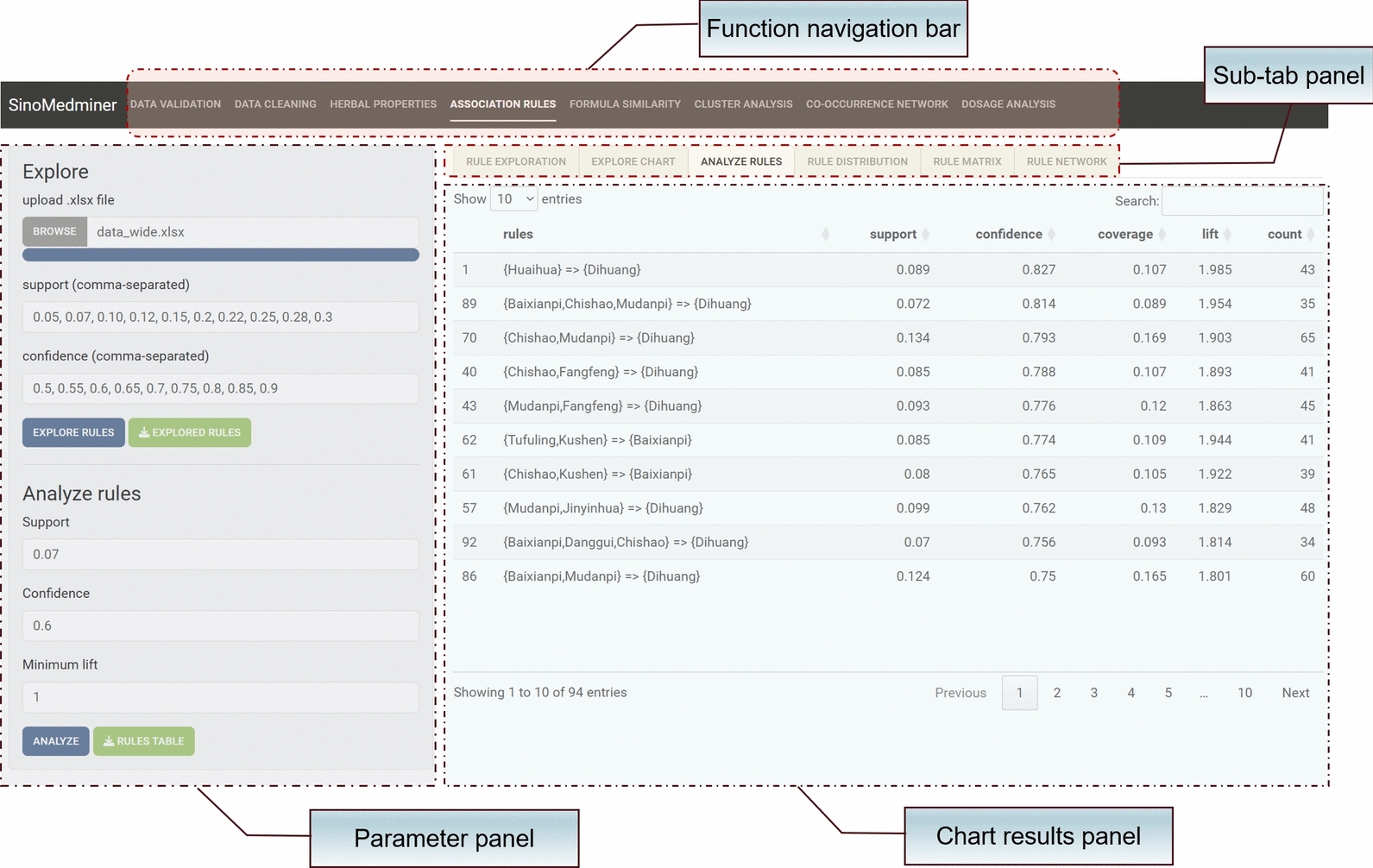
Association rule analysis interface in the SinoMedminer shiny application

The ‘miner_shiny’ function allows direct execution of the Shiny analysis application, enabling users to perform analyses through menu-based interactions without writing any code. By uploading data in the format of the example file, users can quickly generate the corresponding analysis results. The Shiny application consists of 23 panels, providing functionalities such as data cleaning, data transformation, herbal attribute statistics, association rule exploration and analysis, clustering analysis, co-occurrence network analysis, prescription similarity analysis, and dosage analysis. At the top of the application, a navigation bar organizes the panels, with corresponding sub-tabs displayed below. The left sidebar facilitates data interaction and parameter selection, while the lower right section presents tables or graphical results. For instance, Fig. 4 illustrates the visual output of the association rule analysis interface. Additionally, the application can be accessed at http://formulaharmony.com/miner.

### Case application

#### R package with Shiny application.

The SinoMedminer R package has undergone extensive testing across multiple machines and operating systems, demonstrating stable installation and operation on Windows, Mac, and Linux platforms. The current open-source version of the package is available for download from the GitHub repository.

#### Association rule parameter exploration

**Fig. 5:**
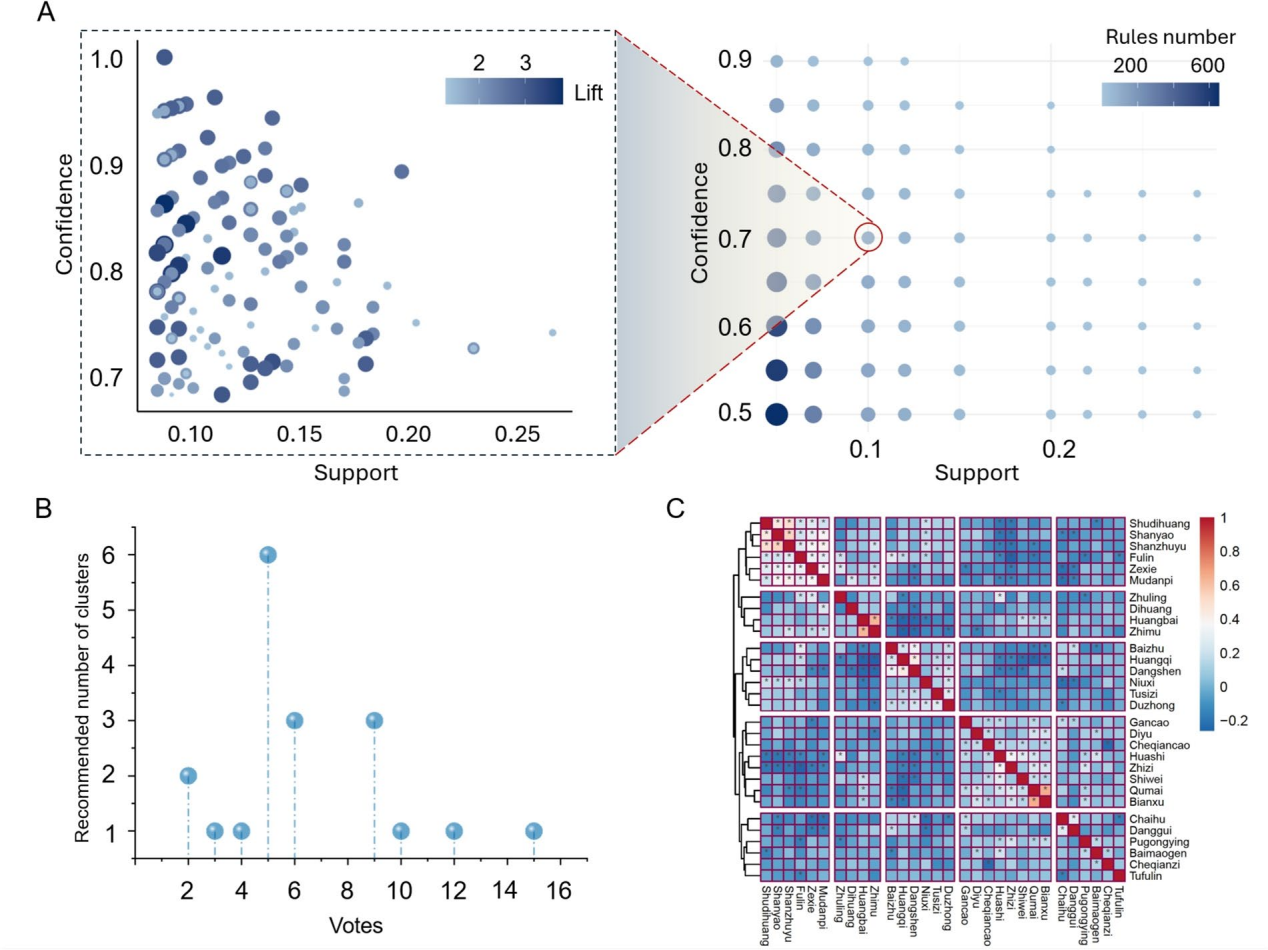
Case application of association rule and clustering. **A** Combination and distribution of association rules with different parameters. **B** Voting recommendation results for the number of clusters. **C** Phi coefficient clustering heatmap. ^*^P < 0.05

Different combinations of confidence and support levels influence the distribution of association rules. However, current research on prescription compatibility mining often presents only the set association rule parameters without demonstrating the results of different parameter combinations. Although the ‘ruleExplorer’ function in the ‘arulesViz’ package can be used to explore the distribution of association rules, it is not effective in providing an intuitive comparison of the differences among various parameter combinations. Consequently, researchers often need to manually test different parameters, reducing analysis efficiency. As shown in the right part of Fig. 5A, we assumed the dataset uses support levels of 0.05, 0.07, 0.10, 0.12, 0.15, 0.2, 0.22, 0.25, 0.28, and 0.3 (10 values), and confidence levels of 0.5, 0.55, 0.6, 0.65, 0.7, 0.75, 0.8, 0.85, and 0.9 (9 values), resulting in 90 different combinations. The ‘explore_rules’ function allows for batch calculation of the number of association rules, the range of confidence and support, and the minimum lift under these 90 combinations. After excluding results affected by two extreme parameter values that lead to ‘-Inf’, 70 combinations remain. Currently, most studies present only the result shown in the right section of Fig. 5A. We recommend that future data mining research provides a more comprehensive view of the results.

#### Cluster exploration

Hierarchical clustering often requires manual determination of the number of clusters. The selection of the optimal number of clusters should consider both clinical and data characteristics, but many current studies lack objective analysis of the number of clusters. First, users can generate dendrograms based on different distance calculation methods using the ‘explore_cluster’ function in the SinoMedminer R package. Then, the ‘k_selection’ function can be used to obtain voting results for the number of clusters recommended by different methods, as shown in Fig. 5B. Among the 20 methods evaluated, the most frequently recommended number of clusters is 5, providing an objective basis for cluster selection, which can then be further refined based on clinical judgment. A Phi-correlation clustering heatmap, based on the characteristics of Chinese herbal prescription data, is shown in Fig. 5C.

#### Formula similarity

**Fig. 6:**
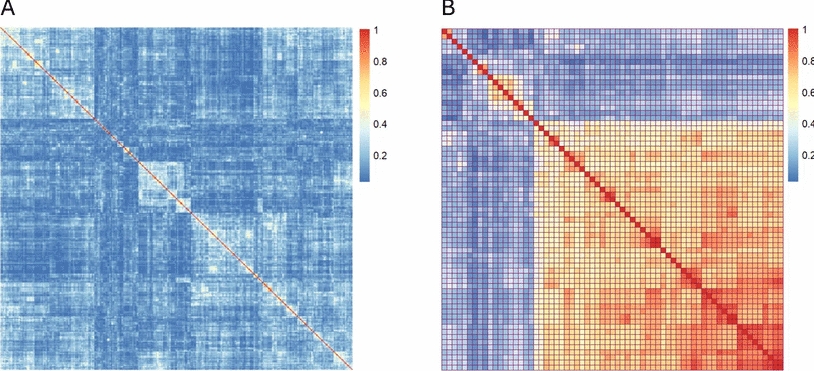
Jaccard coefficient map between different prescription datasets. **A** Prescription dataset from the clinic of National TCM Master Xue Boshou. **B** Prescription dataset from the clinic of Zhang Guangzhong

Formula similarity encompasses two primary aspects: intra-group similarity (similarity between formulas within a single group) and inter-group similarity (similarity between different prescription groups). As depicted in Fig. 6, brighter colors indicate higher similarity between formulas, while darker colors indicate lower similarity. Figure 6A illustrates the outpatient formula dataset of Master Physician Xue Boshu, showing low similarity between formulas within the dataset, which suggests high prescription complexity. Conversely, Fig. 6B illustrates the outpatient formula dataset of Professor Zhang Guangzhong, revealing the presence of numerous formulas with similar compositions within the dataset. Thus, the characteristics of the two prescription datasets differ significantly.

**Fig. 7:**
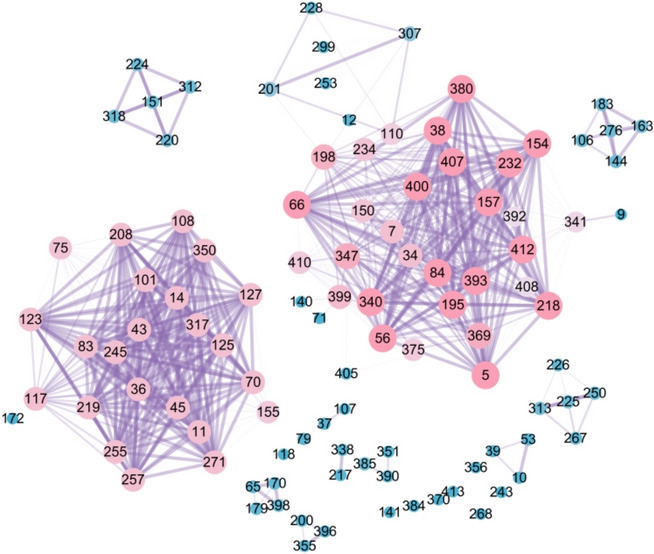
Complex network of prescriptions with Jaccard coefficient > 0.8. The numbers represent prescription IDs

Currently, few studies on formula compatibility have analyzed inter-formula similarity. The ‘calc_jaccard’ function in the R package facilitates the calculation of similarity between any formulas within a single group. If the resulting similarity graph resembles Fig. 6B, further analysis of core formulas within that group can be conducted using network topology. The ‘calc_jaccard’ function returns two data frames, one of which can be used to directly generate a formula network. By applying a prescription similarity coefficient threshold of > 0.8, highly similar formulas can be filtered, and a refined network can be visualized. The degree centrality of each prescription is then calculated, with higher degree values indicating greater importance. As illustrated in Fig. 7, larger nodes representing prescriptions in the network indicate higher frequency of occurrence, reflecting their central position within the group. Thicker edges represent greater similarity between formulas [15]. Intra-formula similarity is a key characteristic of formula groups, and we recommend that future research further explore this aspect.

Furthermore, most current studies on TCM prescription mining focus on a single dataset. However, inheritance is an important characteristic of the TCM field, often necessitating comparisons of prescription variations across different dynasties, inheritors, and regions [16]. The ‘grpSimScore’ function in SinoMedminer can directly calculate the similarity coefficient between two prescription groups.

### Comparison of SinoMedminer with similar tools

**Fig. 8:**
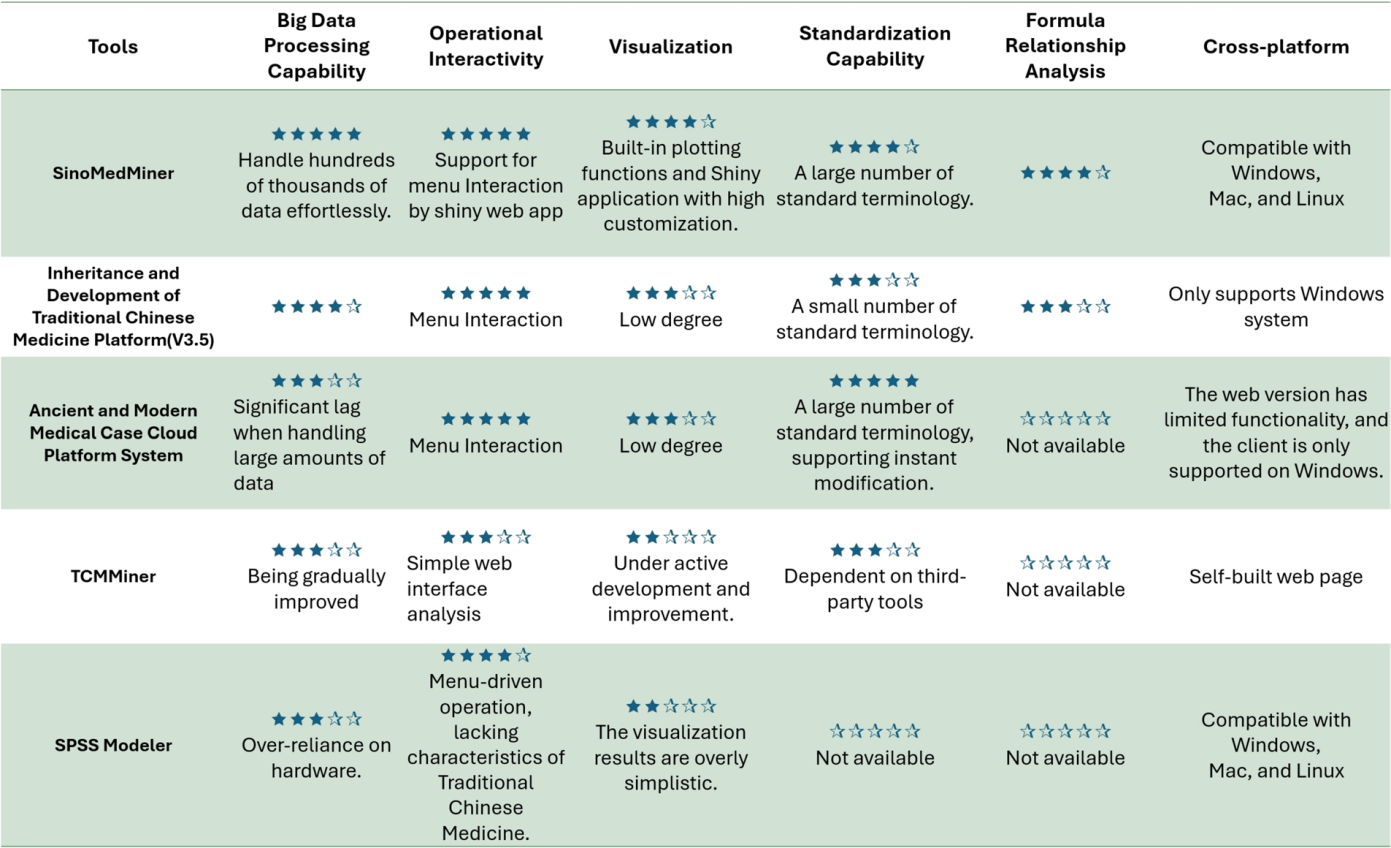
Performance comparison of SinoMedminer with similar prescription data mining tools

The current version of the SinoMedminer R package offers a distinct advantage by leveraging R's powerful big data processing and visualization capabilities while incorporating menu-driven interactive analysis through the Shiny framework [17]. However, because it requires execution of some code via tools such as RStudio, users still need to possess some coding proficiency. In contrast, platforms like the Inheritance Development Platform and the Ancient and Modern Medical Cases Cloud Platform offer more comprehensive software architectures with fully menu-driven operations, resulting in a shallower learning curve compared to SinoMedminer. A comparison of analytical capabilities between similar tools is presented in Fig. 8.

## Discussion

A purely code-based workflow involves numerous variable names, posing challenges for maintenance, sharing, and reproducibility. R packages are core components of the R ecosystem, enabling the development of additional functions and datasets for domain-specific data analysis, visualization, modeling, and application development [18]. R packages encapsulate complex functions and expose them to users through standardized interfaces, simplifying the use of advanced functionalities and enhancing the accessibility of modern data analysis techniques [19]. For example, the survex R package facilitates the interpretation of machine learning survival models, and the oncoPredict R package enables the prediction of drug response and biomarkers in vitro or for cancer patients based on cell line screening data [20, 21]. Currently, no mature R package is specifically designed for analyzing clinical prescription data in TCM. The TCMNPAS platform, developed using the Shiny framework, offers some capabilities for prescription analysis but lacks sufficient analytical depth [22]. By using the SinoMedminer R package, TCM practitioners can rapidly establish a data analysis framework, obviating the need to write foundational code from scratch and allowing them to focus directly on core research questions.

SinoMedminer offers four principal advantages. First, it leverages the efficient data processing capabilities of a programming language to address the performance limitations of currently available software. Second, the inclusion of standardized TCM data within the R package enables users to conveniently standardize their data, addressing the lack of internal datasets in traditional menu-driven software. Third, the Shiny application's menu-driven interface allows TCM practitioners unfamiliar with programming to input and analyze data using SinoMedminer, while those proficient in programming can further enhance their workflow efficiency. Fourth, SinoMedminer is cross-platform, mitigating the challenges faced by some Mac users unable to utilize the Inheritance Calculation Platform or the Ancient and Modern Medical Cases Cloud Platform. Furthermore, the R package's built-in help documentation provides comprehensive documentation and illustrative examples, reducing the barrier for TCM practitioners to use R for data analysis and easing the learning curve associated with R.

SinoMedminer has several limitations. A robust data analysis platform should accommodate files from other platforms, and future research will focus on developing features to convert data files from the Inheritance Calculation Platform into formats compatible with SinoMedminer. Additionally, the current formula similarity calculation does not account for herbal dosages, an area that will be explored in future research.

In conclusion, SinoMedminer has demonstrated promising performance. Future development will focus on creating a Python-compatible SinoMedminer library for enhanced cross-platform compatibility and integrating it with network pharmacology approaches to better elucidate the mechanisms of action of Chinese herbs [23, 24]. Future research will also explore incorporating Chinese medicine omics analysis techniques to further promote the application and development of data science in TCM.

## Data Availability

The datasets used or analyzed during the current study are available from the corresponding author on reasonable request.

## Abbreviations

CNKI: China National Knowledge Infrastructure
ETCM: Encyclopedia of Traditional Chinese Medicine
SPSS: Statistical Package for the Social Sciences
TCM: Traditional Chinese Medicine
TCMNPAS: Traditional Chinese Medicine Network Formulaology and Pharmacology Analysis System
TCMSSD: Traditional Chinese Medical Syndrome Standardization Database
